# Returning to Work Following Low Back Pain: Towards a Model of Individual Psychosocial Factors

**DOI:** 10.1007/s10926-014-9522-9

**Published:** 2014-05-21

**Authors:** Elyssa Besen, Amanda E. Young, William S. Shaw

**Affiliations:** Liberty Mutual Research Institute for Safety, Hopkinton, MA USA

**Keywords:** Return-to-work, Recovery expectations, Work disability prevention, Individual psychosocial factors, Fear-avoidance beliefs

## Abstract

*Purpose* The aim of this paper is to develop and test a model of direct and indirect relationships among individual psychosocial predictors of return-to-work (RTW) outcomes following the onset of low back pain (LBP). *Methods* We utilize secondary analysis of a larger study of adults seeking treatment for work-related LBP with recent onset. In total, 241 participants who completed a baseline survey, a short follow-up survey, and a longer follow-up survey after 3 months were included in our analyses. The participants were required to have LBP with onset of less than 14 days, be 18 years or older, and be fluent in English or Spanish. The analyses utilized structural equation models to test the direct and indirect relationships among the variables and RTW outcomes at 3 months. *Results* Our results indicated a good fit for our model (χ2 = 69.59, *df* = 45, *p* < .05; RMSEA = .05; CFI = .95; WRMR = .61). Pain, catastrophizing, fear-avoidance beliefs, organizational support, and RTW confidence were all found to have indirect relationships with the outcomes. RTW confidence and RTW expectations were found to have direct relationships with the outcomes. *Conclusions* The process of returning to work after an episode of LBP is a complex process involving many interrelated factors. Understanding the relationships among critical individual factors in the RTW process may be important for the treatment and rehabilitation of those with LBP. Results suggest that if injured workers are struggling with fear avoidance, pain catastrophizing and confidence issues, they might benefit from the application of cognitive behavioral therapy techniques.

## Introduction

Low back pain (LBP) is a highly prevalent cause of disability [1] and one of the most expensive health conditions [2], costing Americans approximately $50 billion annually [3]. As much as 70–90 % of the population will experience at least one episode of LBP in their lifetime [4–6], and depending on the definition used, studies have reported that between 24 and 87 % of sufferers have subsequent LBP within a year after their initial episode [7, 8]. Along with personal suffering, LBP can result in decreased productivity and absenteeism [5, 9, 10]. It is also one of the leading causes of lost work time [11, 12]. According to the United States Bureau of Labor Statistics, among work-related musculoskeletal injuries and illnesses resulting in lost time from work, 42 % were back-related conditions that resulted in a median of 7 days of lost work time [13]. As such, there has been interest in examining the factors associated with returning to work after an episode of LBP.

Rather than viewing work resumption as a discrete event, returning to work after an episode of work disability can be viewed as a process that encompasses a series of events, transitions, and phases, and includes interactions with other individuals and the environment. [14]. Consistent with this way of thinking, for this study, the return to work (RTW) process is conceived of as the process workers go through in order to reach, or attempt to reach, their RTW goal (typically a return to their pre-disability work participation). The process is thought of as beginning at the onset of work disability (defined as any restriction to usual work participation, and not necessitating time of work) and concluding when a satisfactory long-term outcome has been achieved.

The biopsychosocial approach has been popular in research on RTW. This approach argues that both biological and psychosocial factors contribute to the development of pain and disability. Two of the main tenets of the biopsychosocial approach are that pain does not directly predict disability outcomes and “Psychosocial factors mediate one’s reaction to injury” (p. 340) [15, 16]. Within the biopsychosocial tradition, there have been numerous studies focused on the predictors of RTW resulting from LBP. Along with biomedical variables, various psychosocial and socioeconomic factors have been explored. There has been a large focus on individual psychosocial factors, although psychosocial factors also include non-individual level variables, such as workplace-level variables like organizational climate and societal-level variables like cultural perception of disability.^1^ Among the individual psychosocial factors that have been found to predict RTW outcomes are: recovery expectations [10, 17–25], fear-avoidance beliefs [10, 22, 26, 27], self-efficacy [26], social support [18, 20, 28–31], and catastrophizing [32, 33].

The majority of the studies of RTW after an episode of LBP focus on direct predictors only as opposed to examining possible indirect relationships among the predictors. Oftentimes predictors are considered in a single model, with one variable being suggested as more important than the other variables. As Campbell et al. [34] point out, a problem with this is the possible conceptual overlap among the psychosocial variables. Nevertheless, exploring the underlying concepts associated with the wide range of factors relating to work disability following a LBP episode does not consider the possibility of indirect relationships among the factors, with one variable exerting its influence on RTW through its relationship with another variable. Building off the biopsychosocial tradition, it is important to explore the potential for indirect relationships among psychosocial factors to better understand the RTW process and why pain level does not directly lead to outcomes.

Research into the indirect relationships among various individual psychosocial predictors of RTW is limited; however, there are reasons to expect the existence of indirect paths. Several studies have proposed mediational paths, where one variable exerts its influence on the ultimate outcome through a relationship with an intermediate variable, which are involved in the development of depression in episodes or chronic instances of pain [35–37]. In addition, studies have examined path models for functional disability [38–41] and transitioning from acute to chronic pain [42]. It is likely that in the context of RTW, indirect paths among individual psychosocial factors also exist. For example, one study found that supervisor response was indirectly related to mental health outcomes after a workplace injury through the relationship with perceived fairness [43]. To examine possible indirect paths, we apply the ABC model which is commonly used in cognitive behavioral therapy (CBT). CBT has been found to be an effective treatment for LBP [44–47] and is frequently used by those treating patients with LBP.

**Fig. 1:**
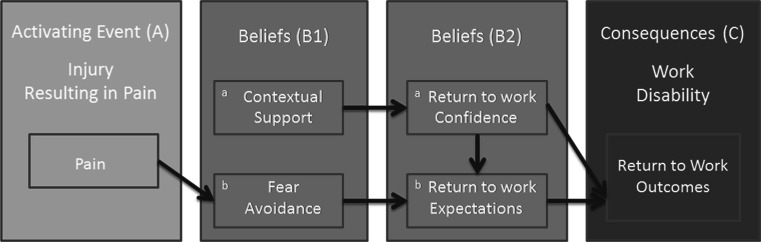
AB(B)C Model of work disability showing the conceptual model of constructs and pathways amongst them

The ABC model suggests that first, there is an activating event (A), which leads to evaluations of the event or beliefs (B), and finally there are consequences (C) of those beliefs that may lead to a specific behavior or outcome [48, 49]. Building off this model, we propose that in the case of RTW following LBP, first there is an activating event (A), in this case the onset of pain.^2^ Next, beliefs (B) are formed based on pre- and post-onset experiences and perceptions. Finally, there are consequences (C), including RTW outcomes, which result from one’s beliefs. To explain this further, in our adaptation of the ABC model (see Fig. 1), we allow for the development of beliefs based on post-onset experiences and perceptions, as well as pre-existing beliefs, and break beliefs into two groups. The first involves beliefs that are present prior to the activating event (B1a), in this case perceptions of contextual support and those that are formed following the activating event which are based on preexisting cognitions (B1b), in this case fear avoidance. The second set involves beliefs that are formed once an injured individual has been able to evaluate his/her situation with regards to work and are influenced by (among other things) the first set of beliefs. These beliefs likely include confidence about one’s ability to RTW (B2a) and RTW expectations (B2b).

Using the ABC model as a conceptual framework for our analysis, our goal was to develop a model of individual psychosocial variables which are characteristics of individuals’ perceptions of their general work environment and specific aspects of their LBP and the impact of that LBP on their lives. We include both direct and indirect relationships involved in the RTW process after seeking treatment for recent onset LBP. In doing so, we tested the model using analyses of secondary data from a sample of individuals experiencing LBP with recent onset who were followed for 3 months to assess RTW outcomes.

## Methods

### Participants

The sample for this study was drawn from a larger study of 496 adults seeking treatment for work-related LBP with recent onset from private medical occupational clinics in Massachusetts, Rhode Island, and Texas. The participants were required to have non-specific sacral or lumbar back pain, with onset of less than 14 days, be 18 years or older, and fluent in English or Spanish. The majority of the participants were employer-referred to the clinics, although some were referred from primary care providers or emergency rooms. Data collection occurred at three time points; the first was during the patient’s initial visit to the clinic, the second was a mean of approximately 7 days later, and the third was a mean of approximately 3 months following the patient’s initial visit. For the current study we focused on the subsample of participants who completed all three assessments (N = 241). The majority of the sample was male (54 %), white (72 %), and non-Hispanic (78 %). They ranged in age from 18 to 63, with an average age of 38 (SD 11.4). Forty-seven percent of the sample had job tenure of less than 2 years and 51 % had more than a high school degree. The majority of the sample (55 %) had an annual income of $15,000–39,999, and worked in blue collar occupations (76 %). Forty-three percent were married at the time of the initial visit. For more information on the larger study, please see the previous work of Shaw et al. [50, 51].

### Procedures

Patients presenting with recent onset LBP at the initial visit to the clinic were informed about the research study and a consent form was provided. If participants consented to participate, they were asked to fill in a 10-page questionnaire that took approximately 10–15 min to complete. The questionnaire asked questions regarding demographics, pain, recovery expectations, and functional capacity. Upon returning to the clinic for a follow-up visit, participants were again asked to complete the 10-page questionnaire. Approximately 3 months after the initial visit, participants were asked to complete a follow-up questionnaire examining pain, functional limitation, and work status. Participants had four choices as to how they would complete the 3-month survey: by conducting a live one-on-one telephone interview with project staff, using a telephone-based interactive voice response service, completing a web-based survey, or returning a paper survey. Participants were given a $30 retail gift card for completing the initial survey, and a $25 payment (in the form of a check) after completing the 3-month follow-up. All study procedures were approved by the institutional review board for the Liberty Mutual Research Institute for Safety.

### Hypothetical Model

In testing the conceptual model proposed in this paper, we use specific individual psychosocial factors. As was previously mentioned, the activating event is an injury that results in pain (A). Our model only focuses on injuries associated with pain that require a person to seek medical treatment and result in some disturbance to his or her work participation. Pain is then thought to lead to fear-avoidance beliefs (B1b). In testing this portion of the model, we utilize aspects of the fear-avoidance model [52–54], which poses that when pain is perceived to be threatening, this leads to pain catastrophizing. Pain catastrophizing refers to an “exaggerated negative orientation towards noxious stimuli” (p. 499) in this case the pain experience [55]. Pain catastrophizing in turn results in pain-related fear of movement and re-injury, known as kinesiophobia, and ultimately avoidance beliefs and behaviors. Previous research examining the fear-avoidance model has found support for the model [56, 57]. Building off this, we examine paths from pain (A), to pain catastrophizing which leads to pain-related fear beliefs (B1b).^3^

In testing the next portion of the model, we apply aspects of the theory of planned behavior [58]. This theory posits that intentions to perform a behavior are predicted by attitudes towards the behavior, by subjective norms, and by perceived behavioral control. In turn, the intentions to perform a behavior, along with perceived behavioral control, predict the actual behavior. In this theory, attitudes towards the behavior refer to an individual’s evaluation of a given behavior. Subjective norms refer to perceived social standards about the given behavior. Perceived behavioral control is similar to self-efficacy and involves an individual’s confidence in their ability to perform the given behavior. Intention to perform the behavior refers to expectations and motivation to perform a given behavior. This theory has been successfully applied to work expectations and outcomes following a musculoskeletal injury [59] and to workers on long-term sickness absence [60]. Appling the Theory of Planned Behavior, we propose that RTW confidence (B2a) is related to perceived behavioral control, pain-related fear beliefs (B1b) are a form of attitudes towards a behavior, RTW expectations (B2b) represent an intention to perform a behavior, and RTW outcomes (C) are the given behavior in this case. Based on this, we expect RTW confidence (B2a), which we define as a person’s confidence in their ability to RTW, and pain-related fear beliefs (B1b) to relate to RTW expectations (B2b), which we define as a person’s prediction of their future work status. RTW expectations are a complex phenomenon encompassing many factors including when a person may RTW, in what capacity a person may RTW, and what functional limitations may be present upon RTW, however in the current study we focus on RTW expectations as expectations for the time (duration) to RTW without limitations. In turn, we expect both RTW confidence and expectations to relate to the RTW outcomes (C).^4^

Moving beyond the Theory of Planned Behavior, we expect contextual support (B1a), which we define as social support both within and outside of work, to relate to RTW confidence (B2a). Having confidence in the ability to complete a behavior is similar to self-efficacy, which is defined as “beliefs in one’s capabilities to organize and execute the courses of action required to produce given attainments” (p. 3) [61]. Previous work has suggested a relationship between social support and self-efficacy, with social support thought to serve to heighten self-efficacy beliefs [62]. In one study, self-efficacy was found to mediate the relationship between social support and adherence to recommendations for heart failure [63]. Along these lines, contextual support in the form of organizational and coworker support may help to bolster one’s confidence in the ability to RTW after an episode of LBP.

In the current study, the activating event (A) and the first set of beliefs (B1) was assessed with data collected during the initial visit. The second set of beliefs (B2) was assessed with data collected during the 7-day follow-up visit. And the consequences (C) were assessed with data collected during the 3-month follow-up interview.

### Measures

#### Activating Event (A)

The activating event was measured as pain using the single item “Please indicate your current level of back pain.” An 11-point scale (from 0 “no pain” to 10 “worst imaginable pain”) was used with higher scores indicating a greater level of pain. Numerical pain rating scales have been shown to be valid and reliable and they have been shown to be sensitive to change in LBP treatments [64, 65].

#### Beliefs (B1)

The first set of beliefs included fear avoidance, assessed as pain catastrophizing and fear-avoidance beliefs, and contextual support, assessed as organizational support and coworker support. Pain catastrophizing was measured using the Pain Catastrophizing Scale [66]. The scale consists of 13 items on a 5-point scale (0 “not at all”, 1 “to a slight degree”, 2 “to a moderate degree”, 3 “to a great degree”, and 4 “All the time”) with higher scores indicating a greater degree of catastrophizing. A sample item includes “I worry all the time about whether the pain will end.” The internal consistency for these items in this study was high (alpha = .95) and the reliability and validity of this scale has been previously validated [55, 66]. Fear-avoidance beliefs were measured using the Tampa Scale of Kinesiophobia [67]. A shorter, 11-item version of this scale was chosen for use in the study [68]. The items were measured on a 4-point scale (1 “Strongly disagree”, 2 “disagree”, 3 “agree”, 4 “strongly agree”), with higher scores indicating greater fear-avoidance. A sample item includes “I can’t do all the things normal people do because it’s too easy for me to get injured.” This measure has been previously validated and had a high internal consistency in this study (alpha = .80) [68]. Organizational support was measured using the an 8-item shortened version of the Perceived Organization Support scale which has been previously validated and used in a study on chronic pain [69, 70]. Items were assessed on a 7-point scale (1 “strongly disagree”, 2 “moderately disagree”, 3 “slightly disagree”, 4 “neither agree nor disagree”, 5 “slightly agree”, 6 “moderately agree”, 7 “strongly agree”) with higher scores representing greater organizational support. A sample item includes “This organization takes pride in my accomplishments at work.” The internal consistency for this measure was .88. Coworker support was measured with the Workplace Friendship Scale [71]. Six items were assessed on a 7-point scale (1 “strongly disagree”, 2 “moderately disagree”, 3 “slightly disagree”, 4 “neither agree nor disagree”, 5 “slightly agree”, 6 “moderately agree”, 7 “strongly agree”) with higher scores signifying greater coworker support (alpha = .84). A sample item includes “I can confide in people at work.” Both the organizational support and coworker support measures focused on global aspects of these constructs and were not meant to assess levels of support related to the specific LBP experience.

#### Beliefs (B2)

The second set of beliefs included RTW confidence and RTW expectations. RTW confidence was measured using the 19-item Return-To-Work Self-Efficacy Scale [50]. Respondents were asked on a scale from 1 (not at all confident) to 10 (totally confident) how confident they were about meeting their job demands, communicating their needs with others, and modifying their work tasks. This scale has been previously validated and had a high internal consistency (alpha = .96) [50]. A sample item includes “How confident are you that you could fulfill all of your duties and responsibilities.” RTW expectations were measured as duration to RTW using the single item “How soon do you expect to be able to resume your normal job without any limitations?” Responses were coded 1 for 0–2 days, 2 for 3–7 days, 3 for 8–14 days, 4 for 15–30 days, 5 for 31–60 days, and 6 for more than 60 days. Scores were reversed so that higher scores indicate more favorable expectations (fewer days to resume normal job).

#### Consequences (C)

Three different RTW outcomes at 3 months were used to measure the consequences: days of absence, days of work limitations, and work status. Days of work absence was measured with the single item “Please estimate the number of days you were absent from work over the past 3 months due to back pain.” Days of work limitation was measured with the single item “Please estimate the number of days you were on modified, alternate, or restricted duty over the past 3 months due to back pain.” Responses to both items were coded 1 for 0 days, 2 for 1–3 days, 3 for 4–7 days, 4 for 8–30 days, and 5 for more than 30 days. Work status was a categorical variable created by combining a single item on whether the respondent was working and a 16-item version of the Work Limitations Questionnaire (WLQ) [72]. For those reporting that they were working at the time of the 3-month follow up, a continuous scale based on the 16-items was created. The distribution was highly skewed due to the large number of participants reporting no work limitations. To address this concern, we created a categorical variable using respondents’ scores on the WLQ coded as 1 for working with no limitations, 2 for working with minor limitations, and 3 for working with major limitations. Using the responses from the question about whether the respondent was working, we coded participants as 4 if they were not working because back pain was preventing them. To verify if the categories follow an increasing pattern of limitations, we examined respondents functional limitations using the Quebec Back Pain Disability Scale [73]. For all groups, functional limitations were significantly different [F(3, 218) = 252.07, *p* < .05] and they increased from groups 1–4. Work status was reverse coded so that higher scores indicate less work limitation.

### Analytic Strategy

All descriptive analyses were conducted using STATA (version 13.0). Means, standard deviations, and correlations for all study variables can be found in Table 1.

**Table 1:** Descriptive analyses and correlations (N = 241)

Variables		Mean (SD)	Range	1	2	3	4	5	6	7	8	9
1	Pain	7.0 (1.9)	0–10	–								
2	Catastrophizing	1.5 (1.0)	0–4	.39***	–							
3	Fear-avoidance beliefs	2.6 (.5)	1–4	.19**	.58***	–						
4	Organizational support	5.2 (1.4)	1–7	.02	−.15*	−.19**	–					
5	Coworker support	4.9 (1.4)	1–7	.11	.06	−.01	.36***	–				
6	RTW confidence	6.5 (2.4)	1–10	−.12	−.13*	−.24***	.34***	.30***	–			
7	RTW expectations	2.9 (1.5)	1–6	−.22***	−.39***	−.32***	.23***	.12	.41***	–		
8	Days of absence	%		.23***	.23***	.21***	−.11	−.04	−.34***	−.19**	–	
	0 days	42										
	1–3 days	20										
	4–7 days	12										
	8–30 days	10										
	30+ days	17										
9	Days of limitation			.00	.09	.17**	−.03	−.02	−.13*	−.28***	.24***	–
	0 days	33										
	1–3 days	10										
	4–7 days	12										
	8–30 days	28										
	30+ days	17										
10	Work status			−.33***	−.37***	−.27***	.26***	.08	.32***	−.42***	−.57***	−.12
	Not working due to pain	14										
	Working with major limitations	17										
	Working with minor limitations	46										
	Working with no limitations	23										

* *p* < .05; ** *p* < .01; *** *p* < .001

#### Structural Equation Modeling (SEM)

**Fig. 2:**
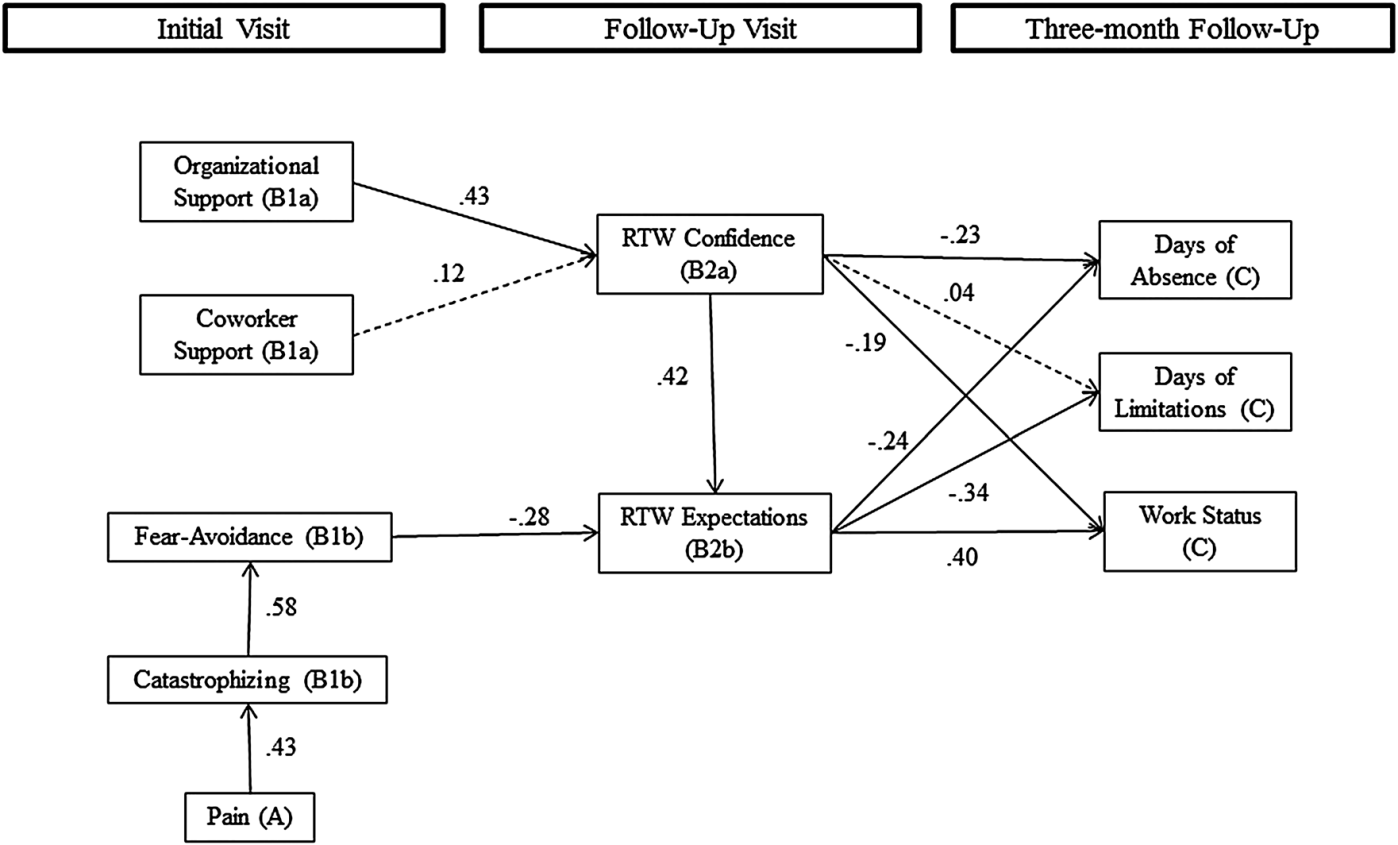
Estimated hypothetical model of psychosocial factors associated with RTW outcomes 3 months following the onset of LBP. Values presented are standardized regression coefficients. *Solid lines* represent paths significant at the *p* < .05 level. *Dashed lines* represent estimated paths that were not significant. Model fit: χ^2^ = 69.59, *df* = 45, *p* < .05; *RMSEA* = .05; *CFI* = .95; *WRMR* = .61. The *boxes* with the respective visits represent the time at which each variable in the boxes below was assessed

Structural equation modeling was implemented to test our hypothetical model using Mplus (version 6.1, Muthén and Muthén, Los Angeles, CA). SEM is a regression-based modeling technique which allows for the simultaneous estimation of both direct and indirect relationships, as well as multiple outcome variables. To test for indirect relationships, the coefficients represent the product of the direct relationship coefficients involved in the indirect path. The significance levels for these coefficients are derived using the delta method [74]. As our outcome variables were ordered categorical variables, we used weighted least squares means and variance adjusted estimation (WLSMV). This method is appropriate for categorical data as it does not require multivariate normality [75]. Oftentimes a full structural equation model includes both a measurement model, which relates observed variables to latent constructs, and a structural model, which relates the latent constructs to other latent constructs. In the current study we are using measures that have been validated elsewhere and since we have a limited sample size for running confirmatory factor models we are treating all variables, including our created latent scales, as observed. In our models, we tested the significance of a set of control variables including age, gender, race, ethnicity, education, income, marital status, and occupational group. We have only included controls reaching significance at *p* < .05. Ethnicity, race, income, and education were retained in the model; however for simplicity, we have omitted these paths from Fig. 2 which shows the estimated model.

#### Model Fit

The SEM model fit was assessed using χ^2^, the Comparative Fit Index (CFI), the Root Mean Squared Error of Approximation (RMSEA), and the Weighted Root Mean Square Residual (WRMR). Using these fit statistics, a good model fit is indicated by a CFI of more than .95, with a maximum of 1, a RMSEA of .06 or lower, and a WMRM of less than 1 [76, 77]. In addition, a non-significant χ^2^ is thought to indicate good model fit, however this statistic is highly sensitive to sample size and so a χ^2^ that is less than twice the size of the degrees of freedom is generally thought to show good model fit [78].

## Results

As can be seen in Table 1, significant correlations were observed among work status at 3 months and all of the predictors with the exception of coworker support. For days of absence, significant correlations were observed with all predictors except organizational support and coworker support. Finally, for days of work limitation, only fear-avoidance beliefs, RTW expectations, and days of absence showed significant correlations.

### Structural Model

Overall, our model supported the hypothetical model (see Fig. 2).^5^ As expected [53, 54], a higher level of pain was positively associated with catastrophizing (.24, *p* < .001), and in turn a higher level of catastrophizing was positively associated with fear-avoidance beliefs (.29, *p* < .001). In addition, pain was indirectly related to fear-avoidance (.07, *p* < .001) through the relationship with catastrophizing. For RTW confidence, only the direct relationship with organizational support was significant (.77, *p* < .001), with higher organizational support being associated with greater RTW confidence. In contrast, there was no direct relationship for coworker support.

Fear-avoidance beliefs (−.76, *p* < .001) and RTW confidence (.24, *p* < .001) both showed significant direct associations with RTW expectations. Greater fear-avoidance beliefs were associated with less favorable RTW expectations, whereas greater RTW confidence was associated with more favorable expectations. Significant indirect relationships with RTW expectations were found for organizational support (.18, *p* < .001), catastrophizing (−.16, *p* < .001), and pain (−.07, *p* < .01). Regarding the outcomes at 3 months, as expected, there were direct relationships for both RTW confidence (−.10, *p* < .05) and RTW expectations (−.19, *p* < .05) with days of absence, with greater RTW confidence and more favorable RTW expectations, respectively, relating to fewer days of absence. In addition, the indirect relationship of RTW confidence with days of absence through the relationship with RTW expectations was also significant (−.05, *p* < .05). Approximately, 31 % of the total relationship between RTW confidence and days of absence due to back pain was indirect. Significant indirect relationships with days of absence were also found for organizational support (−.12, *p* < .001), fear-avoidance beliefs (.14, *p* < .05), and catastrophizing (.04, *p* < .05). For days of limitation due to back pain, the direct relationship with RTW expectations was significant (−.26, *p* < .01), however, only the indirect relationship of RTW confidence through expectations was significant (−.06, *p* < .01), with 79 % of the total relationship between RTW confidence and days of limitation being indirect. Significant relationships with days of limitation were also found for fear-avoidance beliefs (.20, *p* < .05), catastrophizing (.06, *p* < .05), and pain (.01, *p* < .05). Finally, for work status, there were direct relationships with both RTW confidence (.09, *p* < .05) and RTW expectations (.35, *p* < .001) and as with the other outcomes, there was an indirect relationship with RTW confidence (.09, *p* < .001), which accounted for 48 % of the total relationship with work status. Greater RTW confidence and more favorable RTW expectations, respectively, were both associated with working with less limitation at 3 months. Additionally, indirect relationships with working with less limitation at 3 months were found for organizational support (.14, *p* < .001), fear-avoidance beliefs (−.27, *p* < .01), catastrophizing (−.07, *p* < .01), and pain (−.02, *p* < .05).

### Model Fit

Fit statistics indicate that the hypothetical model has a good fit to the data (χ^2^ = 69.59, *df* = 45, *p* < .05; *RMSEA* = .05; *CFI* = .95; *WRMR* = .61). The model explained 32 % of the variance in work status at 3 months, 11 % of the variance in days of work limitation at 3 months, and 15 % of the variance in days of absence due to back pain. Additionally, the model explained 36 % of the variance in fear-avoidance beliefs, 25 % of the variance in RTW confidence, and 37 % of the variance in RTW expectations.

## Discussion

This secondary analysis tested a hypothetical model of the direct and indirect relationships among individual psychosocial factors and RTW outcomes in persons with recent onset LBP. In testing this model, we utilized aspects of the ABC Model, the fear-avoidance model, and the Theory of Planned Behavior [49, 54, 58]. Our results provided support for the hypothetical model. For the portion of the model representing the path from the activating event to the first set of beliefs and building off the fear-avoidance model, we found a path from pain (A), to pain catastrophizing, to fear-avoidance beliefs (B1b). As expected and in line with previous research, an increase in pain was associated with an increase in pain catastrophizing, which in turn was associated with an increase in fear-avoidance beliefs [56, 57]. In our model we found an indirect relationship between pain and fear-avoidance beliefs, where pain was related to fear-avoidance beliefs through pain catastrophizing. This suggests that individuals with a high level of pain, who do not catastrophize about that pain, may be less likely to experience fear-avoidance beliefs which could prevent resuming normal work function. In total, approximately a third of the variance in fear-avoidance beliefs was explained by pain and catastrophizing.

The second portion of our model examined paths from the first set of beliefs to the second set of beliefs. We assessed paths from organizational support and coworker support (B1a) to RTW confidence (B2a). We expected that higher levels of organizational support and coworker support would be related to higher levels of RTW confidence; however we only found support for the relationship with organizational support. In the preliminary analyses, the correlation between RTW confidence and coworker support was significant, but when estimating organizational support simultaneously with coworker support, only organizational support was significant, suggesting that there is shared variance among these variables. Although we did not test it as part of our model, it is possible that organizational support may mediate the relationship between coworker support and RTW confidence with coworker support influencing RTW confidence through its relationship with organizational support. Importantly, the degree to which people feel supported by their organization appears to influence their confidence in returning to work. This is in line with previous research showing that the degree to which individuals are able to modify their work and have accommodating workplaces is related to better outcomes [79–81]. Likely more modifications and accommodations are available in more supportive organizations which will lead to greater confidence in RTW ability.

The final portion of the model tested the path from the first set of beliefs, to the second set of beliefs, and ultimately to the outcomes. Building off of the Theory of Planned Behavior, as expected and in line with previous research, we found that attitudes towards a behavior, represented by fear-avoidance beliefs (B1b), as well as perceived behavioral control, represented by RTW confidence (B2a), influenced intentions to perform a behavior, represented by RTW expectations (B2b), with over a third of the variance in expectations explained by our model [59, 60]. As fear-avoidance beliefs increased, respondents expected to have more time before returning to work, whereas an increase in RTW confidence was associated with expectations of less time before returning to work. RTW expectations in turn influenced behavior, in this case returning to work after injury, represented by the 3 month outcomes (C). For all three outcomes, the relationship with RTW expectations was significant. This is in line with previous research suggesting that recovery expectations are a strong predictor of work outcomes [23, 82]. An increase in RTW expectations was associated with fewer days of absence, fewer days of limitation, and working with fewer limitations.

According to the Theory of Planned Behavior, perceived behavioral control plays a critical role in outcomes involving both direct and indirect relationships through intentions [58]. We found partial support for this. RTW confidence, which represented perceived behavioral control in our model, had significant indirect relationships with all of the outcomes, but there were only direct relationships for days of absence and work status. For the indirect relationships, our findings suggest that as workers’ confidence in returning to work increases, their expectations for returning sooner also increase, and ultimately work outcomes are more favorable. For the direct relationships, aside from the impact on RTW expectations, having confidence in the ability to RTW is related to fewer days of absence and working with fewer limitations. A possible explanation for the lack of direct relationship between RTW confidence and days of work limitation is that some workers may feel very confident about returning to work because many accommodations are available and so it would not be critical to go back without any limitations. In contrast, other workers may feel very confident about being able to go back to work because they have no limitation in their ability to work. For the former group, this could result in a great number of days of working with limitations, while for the latter group, this could result in a smaller number of days of working with limitations. When these two groups are combined, the results may cancel each other out. Overall, our model captured a significant amount of the variance in all three work outcomes, ranging from 10 to 30 %.

### Implications

There are several practical implications of our findings for interventions targeted at LBP. First, our model showed that pain is indirectly related to outcomes through its relationship with other factors. This is in line with one of the tenets of the biopsychosocial model proposing that pain does not directly lead to work disability [15, 83]. Our findings suggest that simply having a high level of pain will not necessarily result in poor outcomes; instead it may be important to identify individuals who experience fear-avoidance processes as a result of pain, specifically those who enter the spiral of catastrophizing about pain, and then avoiding activity for fear of more pain or subsequent injury. From an intervention perspective, targeting this group of individuals and helping them to readjust their catastrophic thinking could result in more positive RTW outcomes. Empirical support for the potential effectiveness of this approach comes from previous work that has shown that an intervention aimed at minimizing pain catastrophizing and fear of movement was related to higher RTW after whiplash injury [84].

Second, our model illustrated the role of contextual support. The findings suggest that organizational support may be an especially important factor in promoting RTW confidence. Previous research has found that organizations play a key role in assisting successful and timely RTW [85]. Furthermore, organizations offering RTW programs may account in part for the successful returns [85]. Conversely, injured workers who perceive their organizations to be unsupportive may be at risk of less favorable RTW outcomes. Findings suggest that the mechanism through which organizational support influences outcomes may be employees’ development of confidence in their ability to return successfully.

Third, our model addressed some of the factors related to the formation of RTW expectations. While research has shown the importance of recovery expectations [10], relatively little research has focused on the development of the expectations. One exception is a study that found that perceived uncertainty, which involves perceptions of control over RTW, is related to the development of expectations [82]. This finding is consistent with our model which focused on RTW confidence as a precursor to RTW expectations. However, our model added fear-avoidance beliefs as a factor in the development of expectations as well. It has been suggested that modifying recovery expectations can help to speed the recovery process [86]. Based on our findings, interventions aimed at improving workers’ confidence about returning to work and reducing fear-avoidance beliefs may serve to foster more favorable expectations with regards to the duration of expected RTW.

Finally, our model was informed by the ABC model which is commonly used in cognitive behavior therapy (CBT), which has been used effectively in the treatment of back pain [44–47]. Our model provides insight as to what should be targeted when using CBT with the aim of improving RTW outcomes for suffers of LBP. Essentially, CBT aims to address maladaptive ways of thinking which lead to bad behavioral choices, and ultimately poor outcomes [87]. Informed by the current findings, it can be suggested that CBT may effectively be used to help sufferers of LBP modify maladaptive thinking, in this case pain catastrophizing and fear-avoidance beliefs, to promote confidence and coping skills, ultimately leading to more positive expectations and outcomes.

### Strengths and Limitations

The current study had several strengths. We tested a theoretically driven model of the relationships among individual psychosocial factors that are thought to impact RTW outcomes. In testing this model, we utilized SEM which allowed for the testing of direct and indirect relationships simultaneously. The secondary data used in this study had a longitudinal component to it with data collected at three points during the RTW process. This helped to disentangle some of the temporal relationships among the factors. Despite these strengths, there were several limitations that also need to be acknowledged when considering the study findings. Although the longitudinal component was a strength for certain aspects of the model, there were still several relationships that were estimated cross-sectionally. It is a common practice to estimate mediational models using cross-sectional data, but it is more appropriate to estimate these types of relationships with longitudinal data, as a result, our analyses are not able to imply causation and must be interpreted cautiously as they are reflective of cross-sectional associations. All of the data were also self-report. While this makes sense for many of the individual psychosocial variables, it was difficult to know the true extent of lost work time due to back pain. There was no information about whether participants were out of work when visiting the clinics, or when time off work was taken. This means that for participants reporting 10 days off of work, it is unclear if those 10 days were sequential, or spread out over a 3 month period, or if those days occurred immediately following the initial visit, or later in the RTW process. It is possible that the timing of work absence has implications for belief development and ultimately, RTW outcomes.

The data used in the study were from a larger study and was not collected with the goal of testing our conceptual model. Another limitation is that we needed to select the measures from the larger study that most closely aligned with the theoretical constructs in our model. This resulted in cases where the measures may not have been ideal. Most notably, only a single item of RTW expectations, specifically duration until RTW, was included in the larger study. Although studies have assessed RTW expectations as duration until RTW [18, 88], RTW expectations are a complicated construct that go well beyond the simple duration until RTW [82]; and thus, our findings must be interpreted with this in mind. Along these lines, there are several individual psychosocial factors that we were unable to include in our model, such as perceived uncertainty which is a key aspect in the formation of RTW expectations [82], and perceptions of social support outside of work. Additionally, work engagement levels may be an important contextual factor relating to one’s intentions to RTW which we were unable to include in our model. Similarly, there were limited personality factors which may play an important role in the formation of fear-avoidance beliefs. Also, we built off the Theory of Planned Behavior; however we had no measure of subjective norm, which likely plays a role in the formation of RTW expectations.

Although our model accounted for a significant amount of the variance in the 3-month outcomes, there was still a large amount of unaccounted variance, suggesting that there are several factors omitted from our model. In this study, we focused solely on individual factors and thus there are factors missing from our model that are related to the larger environmental context in which work disability occurs, such as workplace, family, social systems and societal factors that are major contributing factors in the biopsychosocial approach, that directly interact with individual psychosocial factors, and that likely account for a substantial amount of variance in RTW outcomes [16]. For example, based on past research it may be suggested that workplace and employment related factors such as workplace fairness, benefits and compensation, the availability of work accommodations, and flexibility of schedules may also impact the amount of time a person is able to take off work [89, 90]. In addition, characteristics of the economy/labor market may also exert an influence [91]. Also, based on the large amount of unexplained variance, it is likely that interventions that may be derived from this model would not be appropriate for all injured workers. The current study was not designed to identify meaningful cutoffs for which individuals may benefit from different types of interventions, such as those targeted at catastrophizing, but future research may seek to address this topic.

We limited our model to focus on testing relationships based on the specific theories we described in the paper. There are additional paths which may have been significant that we did not test. For example, we did not specify a direct path from organizational support to RTW expectations, but in addition to the indirect path we found, there may also have been a direct path. Due to restrictions based on the sample size, we were unable to test the full measurement model which may have introduced measurement error into our estimates. We used previously validated measures which may help to alleviate these concerns. Despite this, future research should aim to replicate the model tested here with a larger sample size, including the measurement model for the appropriate latent constructs.

Finally, the sample for this study was a convenience sample of workers seeking treatment for low back pain. The sample was not random and as a result there was bias introduced. The participants in this study may have represented those with the most severe injuries as they were all seeking treatment. Our sample was limited to low back pain and it is possible that the mechanisms uncovered in this study may be applicable for other diagnoses. Also, many of the workers were referred to the clinics by employers suggesting that they were seeking treatment for a work-related injury which may limit the generalizability of our findings to workers with low back pain from non-work injuries. Future research should aim to replicate this model for diagnoses other than low back pain and in more generalizable samples.

## Conclusions

Returning to work after an episode of LBP is a complex process involving many interrelated factors. Pain, fear-avoidance, contextual support, RTW confidence, and RTW expectations were all found to directly, or indirectly relate to RTW outcomes in workers suffering from LBP. Future work may focus on expanding the model presented here to include additional individual and non-individual level psychosocial factors, as well as biological and environmental influences. Findings suggest that patients of those working to improve RTW outcomes might benefit from the application of CBT techniques if they appear to be struggling with fear avoidance, pain catastrophizing and confidence issues.

## Footnotes

1 In this paper, we are limited in our focus of psychosocial factors specifically to individual-level factors, however the biopsychosocial approach includes non-individual level psychosocial factors as well which have been shown to be important factors in RTW.

2 In our model, we use pain as the activating event, however, activating events may actually be the interaction of many factors, rather than a single event. It is possible that the experience of pain alone would not always be an activating event, rather it may be the interaction of the experience of pain with the demands of a particular job for example, which cause a person to evaluate the activating event. In order to be considered an activating event, there must be some occurrence which leads a person to evaluate the occurrence, ultimately resulting in a specific outcome. Thus, in our model, we are using pain as a proxy for this, as we believe that there must be some experience of pain to trigger an evaluation.

3 The paths in this section of the model are estimated with cross-sectional data, where all measures were collected at one time point. It is more appropriate to test these relationships with longitudinal data, however in the current study we are unable to do that and thus cross-sectional data is used. For this reason, we are unable to assess true causation, and the results should be interpreted with this in mind.

4 Note: Unfortunately in our secondary data, there is no measure of subjective norm available and so we will be unable to apply that portion of the theory of planned behavior.

5 Standardized coefficients are presented in Fig. 2. Unstandardized results are presented in text.
